# Nerve fibre morphometry with transmission electron microscopy: Application of the nucleator probe in ImageJ^✰^

**DOI:** 10.1016/j.mex.2023.102085

**Published:** 2023-03-01

**Authors:** Punit Kumar, Saroj Sharma, Charanjeet Kaur, Indra Pal, Daya Nand Bhardwaj, Tapas Chandra Nag, Tara Sankar Roy, Tony George Jacob

**Affiliations:** a Department of Anatomy, All India Institute of Medical Sciences, New Delhi, India; b Department of Anatomy, Dr. Baba Saheb Ambedkar Medical College & Hospital, Delhi, India; c Eaton-Peabody Laboratories, Massachusetts Eye and Ear, Dept of Otolaryngology-Head and Neck Surgery, Harvard Medical School, Massachusetts Eye and Ear, Boston, MA, United States; d Department of Neurobiology School of Medicine, University of Pittsburgh, Pittsburgh, PA, United States; e Department of Forensic Medicine & Toxicology, All India Institute of Medical Sciences, New Delhi, India; f Department of Anatomy, North DMC Medical College & Hindu Rao Hospital, New Delhi, India

**Keywords:** Axon, Myelin, Stereology, axe, axon, CN, cochlear nerve, DDSA, Dodecenyl Succinic Anhydride, DDW, double distilled water, DMP-30, 2,4,6- Tri (dimethylaminomethyl) Phenol-30, IAM, internal acoustic meatus: M, myelin, MNA, Methyl Nadic Anhydride, PB, phosphate buffer, RT, room temperature, Application of the nucleator probe with ImageJ

## Abstract

Stereology and semiautomated binary image histomorphometry are two common methods used for morphometry of nerve fibres. Nucleator probe can be used for the estimation of morphometric parameters like diameter, perimeter, area and volume of a structure that is approximately either a circle or a sphere. In this study, we estimated these parameters with the help of ImageJ software on calibrated transmission electron micrographs. We procured samples of the cochlear nerve (CN) during winter months, within 6-12 hours of death, to reduce post-mortem autolytic changes. The temporal bones containing the CN were fixed by immersion in chilled paraformaldehyde. After dissecting out from the petrous part of the temporal bone, the CN were osmicated and processed for embedding in resin. From the resin blocks, silver coloured (70 nm) ultrathin sections were cut and picked on 300-mesh copper grids, stained with uranyl acetate and lead citrate and viewed under Tecnai G^2^-20 transmission electron microscope. The transmission electron micrographs had scale bars embedded into them by the software at the time of imaging, and the morphometric parameters of randomly selected nerve fibres were measured using the ImageJ software. The ImageJ software could become a low-cost and dependable tool for nerve fibre morphometry.

• Nucleator probe is used for the estimation of morphometric parameters like diameter, perimeter, area or volume
• Morphometric parameters were estimated by the ImageJ software on calibrated transmission electron micrographs
• The ImageJ software could become a low-cost and dependable tool for nerve fibre morphometry

## Graphical abstract

**Figure:**
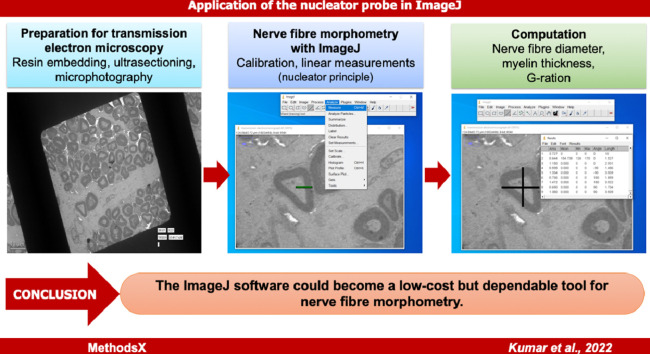

Specifications table

**Table:** 

Subject Area	Neuroscience
More specific subject area:	Nerve fibre morphometry
Method name:	Application of the nucleator probe with ImageJ
Name and reference of original method:	[2,6]
Resource availability:	https://imagej.nih.gov/ij/download.html

**Method details**

## Background

Age-related alterations in the peripheral nerves can be studied by estimations of the number of nerve fibres, their diameters (total and of axons alone) and the thickness of their myelin sheath. These measurements require reliable and efficient methods. The most reliable method used for the evaluation of nerves is unbiased design-based stereology [1] and semiautomated binary image histomorphometry [3,9]. Hunter et al. [4] found that stereological estimations with light microscopy resulted in higher than expected values for nerve fibre and axon diameter than what is seen with histomorphometry and manual counting with the ImageJ software. They proposed that histomorphometry has potential advantages over stereology when stereology is limited to light microscopy alone.

The probe that is used to make these estimations is a local probe called the nucleator [2,5]. Unlike other unbiased probes, the nucleator assumes that the cross-section of particle of interest is roughly circular and employs the summation of the measured radii from an apparent centre and then utilizes the formulae related to the circle to estimate the circumference, area and volume (2πr, πr^2^, 4/3πr^3^), respectively [2]. The truth is that the profiles of nerve fibres are rarely circular; they are more often irregular than circular (Fig. 5 in [4]). Probably, the overestimations found by Hunter et al. [4] on light microscopic images may be minimised by increasing the number of isotropic rays employed in the nucleator probe, though it will make estimations cumbersome for the user [4]. Another limitation that may have affected the results is related to the poor resolution of the light microscopic images. The finer details of the nerve fibres, their boundaries and myelin sheath are best observed by transmission electron microscopy [8]. Therefore, in this study, we have measured the dimensions of nerve fibres on transmission electron micrographs using isotropic rays by adapting the ‘straight line’ tool available in the ImageJ software on the basis of the principle of the nucleator probe [2]. The freely available ImageJ software could become an inexpensive and reliable tool for morphometry at centres that do not have the resources to purchase expensive software for image analysis.

## Materials and reagents

1. Human cochlear nerve (CN)
2. Paraformaldehyde (30,525- 89- 4, Thermo Fisher Scientific India Pvt. Ltd. Mumbai)
3. Glutaraldehyde (G002, TAAB Laboratories Equipment Ltd, England, UK)
4. Osmium Tetroxide (O001, TAAB Laboratories Equipment Ltd, England, UK)
5. Sodium Dihydrogen Orthophosphate Dihydrate (13,472- 35- 0, Thermo Fisher Scientific India Pvt. Ltd. Mumbai)
6. di -Sodium Hydrogen Orthophosphate Dihydrate (10,028- 24- 7, Thermo Fisher Scientific India Pvt. Ltd. Mumbai)
7. Acetone (67 - 64- 1, Thermo Fisher Scientific India Pvt. Ltd. Mumbai)
8. Dry acetone (prepared by adding anhydrous copper sulphate)
9. Toluene (108–88–3, Merck Specialities Private Limited, Mumbai)
10. Araldite CY212 Resin (TAAB Laboratories Equipment Ltd, England, UK)
11. Dodecenyl Succinic Anhydride (D025, TAAB Laboratories Equipment Ltd, England, UK)
12. Methyl Nadic Anhydride (M012, TAAB Laboratories Equipment Ltd, England, UK)
13. 2,4,6- Tri (dimethylaminomethyl) Phenol-30 (DMP-30) (D022, TAAB Laboratories Equipment Ltd, England, UK)
14. Embedding mould (105, Ted Pella, Inc, USA)
15. Toluidine Blue O (SD211, TAAB Laboratories Equipment Ltd, England, UK)
16. Uranyl Acetate EM (U001, TAAB Laboratories Equipment Ltd, England, UK)
17. Lead Citrate AR (LADD Research Industries, Inc. Vermont, USA)
18. 300-mesh copper grids (GG007/C, TAAB Laboratories Equipment Ltd, England, UK)

### Removal of brain to expose the nerve

After obtaining appropriate clearance from the Institute Human Ethics Committee (Ref. No.: IEC/NP-89/2014 RP-36/2014), human temporal bones were collected from the mortuary.

A bone saw was used to remove the bony skull cap of the cadavers during autopsy. The dura mater covering the superolateral surface of the cerebral hemisphere was removed. The temporal lobe of cerebral hemisphere was retracted slightly backwards to visualize the brainstem. The cranial II to VI nerves were cut with a sharp scalpel blade and the VII and VIII nerves were visible entering the internal acoustic meatus. The VII and VIII nerves were cut at that level and the remaining cranial nerves, blood vessels were cut to detach the brainstem from the base of the skull. The medulla oblongata was cut by a transverse incision at the level of upper border of C1 vertebra in the posterior triangle of the neck. The brain was removed gently from the cranial cavity. The apical portion of the petrous part of the temporal bone, along with the vestibulocochlear and facial nerves in the internal acoustic meatus, was carefully chiselled out after removing the brain from the cranial fossae. The bones with the nerves were fixed by immersion in chilled 4% paraformaldehyde made in 0.1 M phosphate buffer (PB, pH 7.4).

### Dissection of the CN

**Fig. 1:**
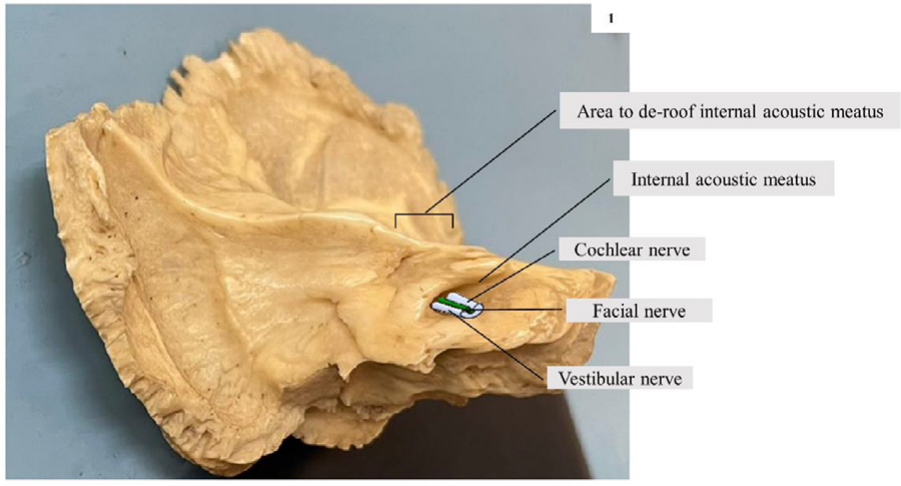
A left temporal bone with its petrous part, position of the cochlear nerve (CN), vestibular and facial nerves at the internal acoustic meatus (IAN), and the area of bone to de-roof the IAM to expose the CN within the IAM.

**Fig. 2:**
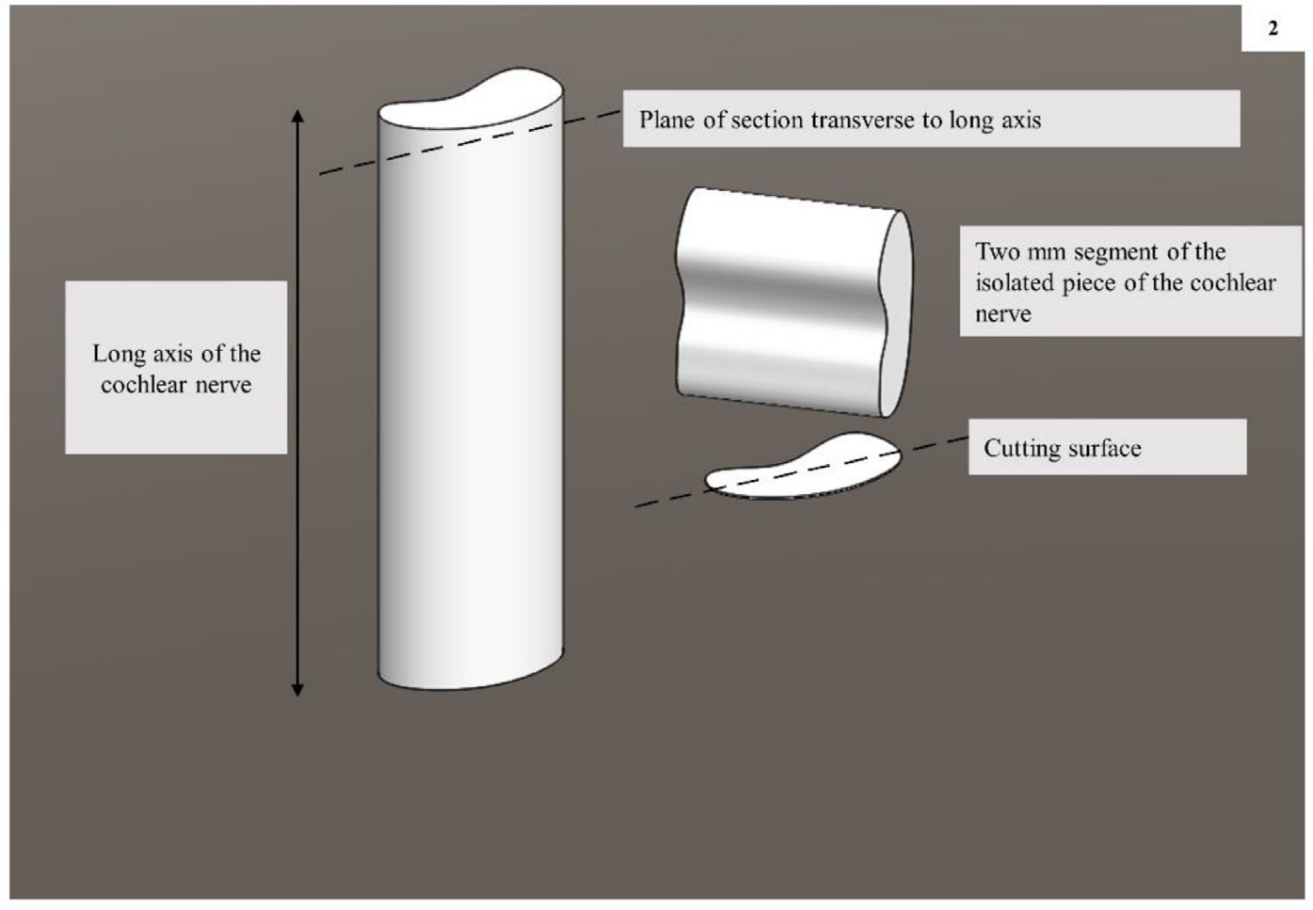
Schematic diagram representing the long axis of the cochlear nerve (CN), plane of sectioning- transverse to the long axis, segment of the isolated piece of the CN used for further processing, and making araldite blocks and the cutting surface.

The petrous part of the temporal bone (Fig. 1**)** was fixed in a bone holder and the internal acoustic meatus (IAM) was de-roofed with a diamond drill (EC300/55,958, Medtronic Xomed, Inc. Florida, USA) in order to expose the CN within the IAM. The CN was cut in a plane that was transverse to its long axis, as close as possible to its exit from the cochlea, with a sharp razor blade. Segments (about 2 mm length) of the isolated pieces of the nerve were transferred into chilled fixative composed of 2% paraformaldehyde and 2.5% glutaraldehyde in 0.1 M PB for 48 h. The nerve segments were osmicated, dehydrated and processed for making resin blocks. In the resin blocks, the nerve segments were placed in such a manner that the cochlear end of the CN remained as the cutting surface (Fig. 2**)**.

### Steps of tissue processing

1. **Primary fixation-** The 2 mm long segment of the CN was immediately immersed in chilled fixative (4 °C) composed of 2% paraformaldehyde and 2.5% glutaraldehyde made in 0.1 M PB for 48 h.
2. Washed three times in 0.1 M PB, pH 7.4 for 15 min each, at 4 °C.
3. **Secondary fixation-** 1% osmium tetroxide in 0.1 M PB (pH 7.4) for 1 hour at 4 °C.
4. Washed three times in 0.1 M PB, pH 7.4 for 15 min each, at 4 °C.
5. **Dehydration-**

i. 30% acetone in double distilled water (DDW)- 15 min at 4 °C.
ii. 50% acetone in DDW- 15 min at 4 °C.
iii. 70% acetone in DDW- 15 min at 4 °C.
iv. 90% acetone in DDW- 15 min at 4 °C.
v. 100% acetone – 15 min x 2 at 4 °C.
vi. Dry acetone – 15 min at 4 °C.
vii. Dry acetone - 15 min at room temperature (RT).

6. **Clearing-**

Toluene – two changes for 30 min each at RT.

7. **Infiltration-**

i. Toluene + Resin (3:1) - 1 hour at RT.
ii. Toluene + Resin (2:2) - Overnight at RT.
iii. Toluene + Resin (1:3) - 1 hour at RT under vacuum (380 mm. Hg).
iv. Pure resin- 2 h at 45 °C under vacuum (380 mm. Hg).

**Fig. 3:**
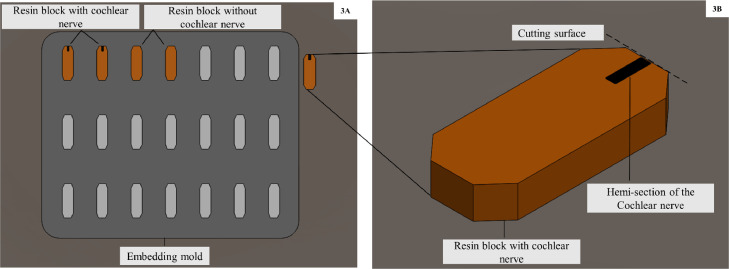
**A:** Schematic diagram of the embedding mould containing polymerised resin with the hemi section of the cochlear nerve (CN). Mould spaces filled with resin without any tissue in it (blank blocks) can be used to check completion of solidification. **B:** Schematic diagram of resin block illustrating cutting surface of the CN.

8. **Embedding-** with pure resin in embedding mould at RT and relative humidity less than 50%.
9. **Polymerisation**- molds were kept at 50 °C for 1 day and then at 60 °C until solidification of resin was completed. To check completion of polymerisation, mould spaces filled with only resin without any tissue in it was used (blank blocks) (Fig. 3).

**Preparation of resin-**

**Table:** 

Araldite CY212 Resin	25 ml
Dodecenyl Succinic Anhydride (DDSA EM)-D025	23.2 ml
Methyl Nadic Anhydride (MNA)-M012	1.8 ml
2,4,6- Tri (dimethylaminomethyl) Phenol-30 (DMP-30)- D022	0.9 ml
Total volume	50.9 ml

**Ultramicrotomy and staining for light microscopy-**

**Fig. 4:**
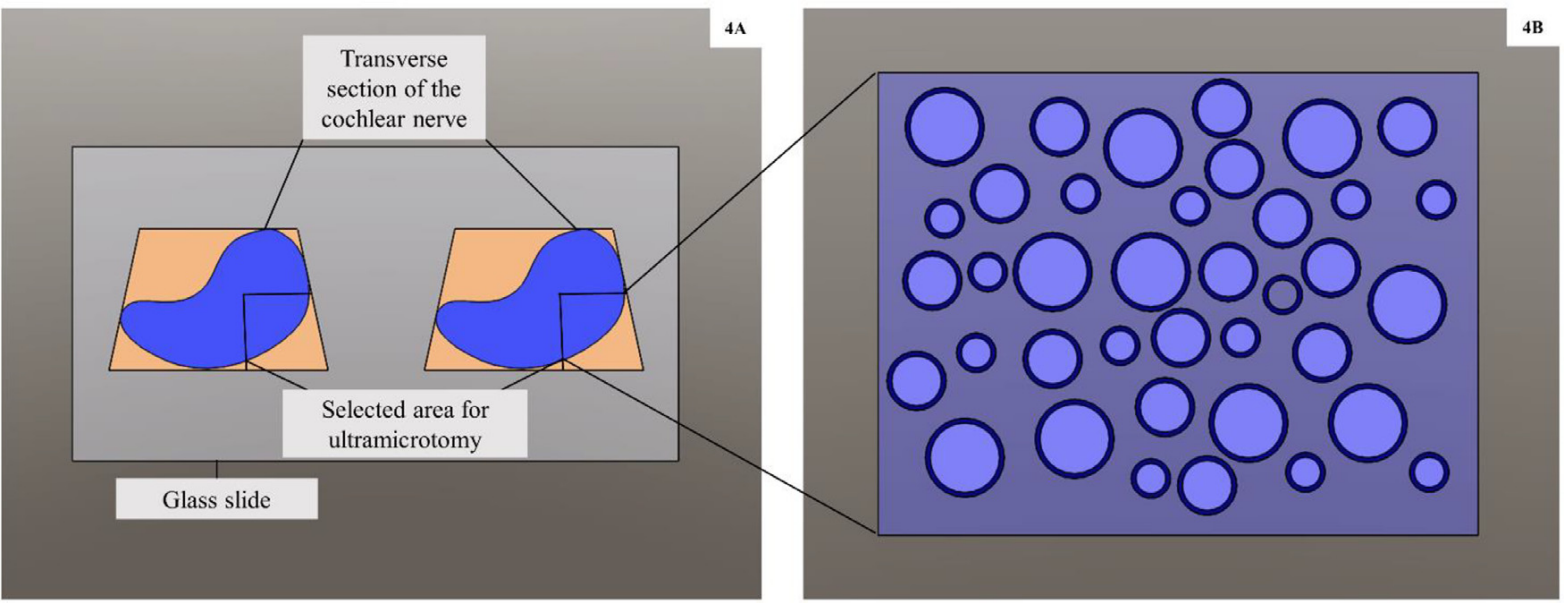
**A:** Schematic diagram of transverse semithin resin sections of the cochlear nerve (CN) collected on a glass slide and stained with aqueous toluidine blue for light microscopic examination. **B:** Magnified view of a representative selected area of the CN for ultramicrotomy.

1. Trimmed and sectioned the resin blocks transversely at 0.5 μm thickness (semithin section) on an ultramicrotome (Ultracut UC7, Leica Microsystems, Vienna, Austria).
2. Put a few sections on a drop of distilled water pipetted onto a clean glass slide and affixed it on the slide by warming it on a hot plate at 80 °C.
3. Added a few drops of aqueous toluidine blue (1% solution) on sections placed on the hot plate for 30 s.
4. Washed the slide with distilled water and dried it.
5. Under a light microscope, selected the area of interest for further trimming and ultrathin sectioning of the blocks (Fig. 4).

**Ultramicrotomy and staining for transmission electron microscopy-**

1. Cut silver coloured (70 nm) ultrathin sections from the resin blocks on an ultramicrotome (Ultracut UC7, Leica Microsystems, Vienna, Austria).
2. Picked these sections on to 300-mesh copper grids.
3. Stained the sections on the grids with aqueous uranyl acetate and alkaline lead citrate

i. Centrifuged the above solutions before use.
ii. Kept a parafilm inside the Petri dish and put 50 µl of uranyl acetate with micropipette on the parafilm.
iii. Floated the grid upon the droplet with the section facing down.
iv. Covered the Petri dish and added one extra black cover upon it to protect the grids bearing the section from light.
v. Kept for 10–15 min.
vi. Washed each grid with double distilled water and dried on a filter paper.
vii. Put small droplet of lead citrate on parafilm kept in a Petri dish. This step should preferably be performed in a hood with laminar flow. This prevents atmospheric carbon dioxide from reacting with lead citrate and forming precipitates of lead carbonate on the grids.
viii. Placed the grid on the droplet with the section facing down.
ix. Stained for 5–10 min.
x. Washed each grid briefly in 0.02 M sodium hydroxide and then in double distilled water twice.
xi. Dried the grids in air.

4. Viewed the grids under the transmission electron microscope (FEI Company, Netherlands).

### Nerve fibre morphometry

1. Scanned the sections of the nerve on Tecnai G^2^–20 transmission electron microscope (FEI Company, Netherlands).
2. Captured the transmission electron micrographs using the Digital Micrograph software (Gatan, Inc).
3. Transferred the captured micrographs onto a personal computer in which ImageJ software (National Institutes of Health, Bethesda, Maryland, USA) was already installed.
4. Opened the ImageJ software (Fig. 5)

**Fig. 5:**
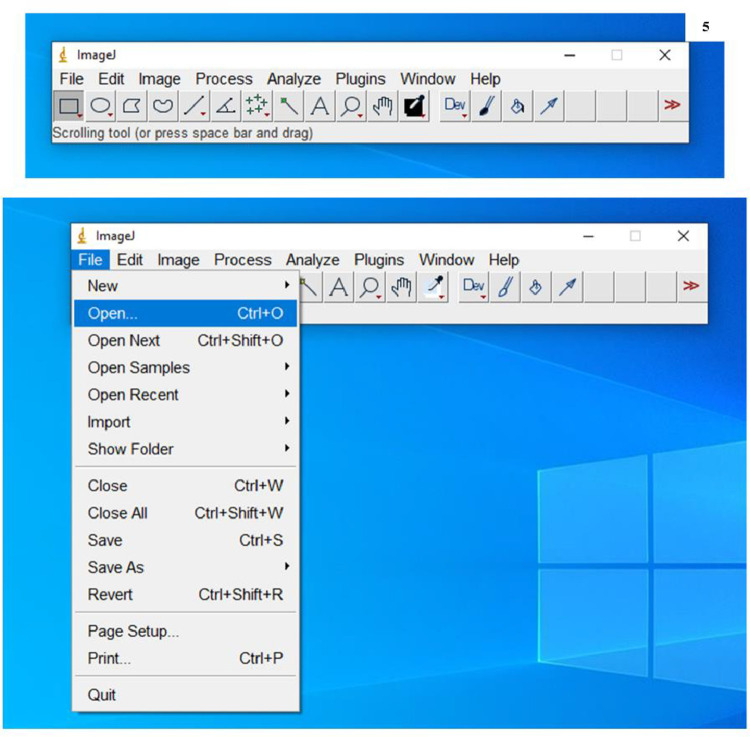
Screenshot explains opening of the captured transmission electronmicrograph file in the ImageJ software.
5. Opened the micrographs in ImageJ software (File>Open>Select image) (Fig. 5)
6. Selected the magnifying glass (digital zoom) from the tool bar in the ImageJ window (Fig. 6A) and left-clicked on the image to magnify the scale bar (Fig. 6B)

**Fig. 6:**
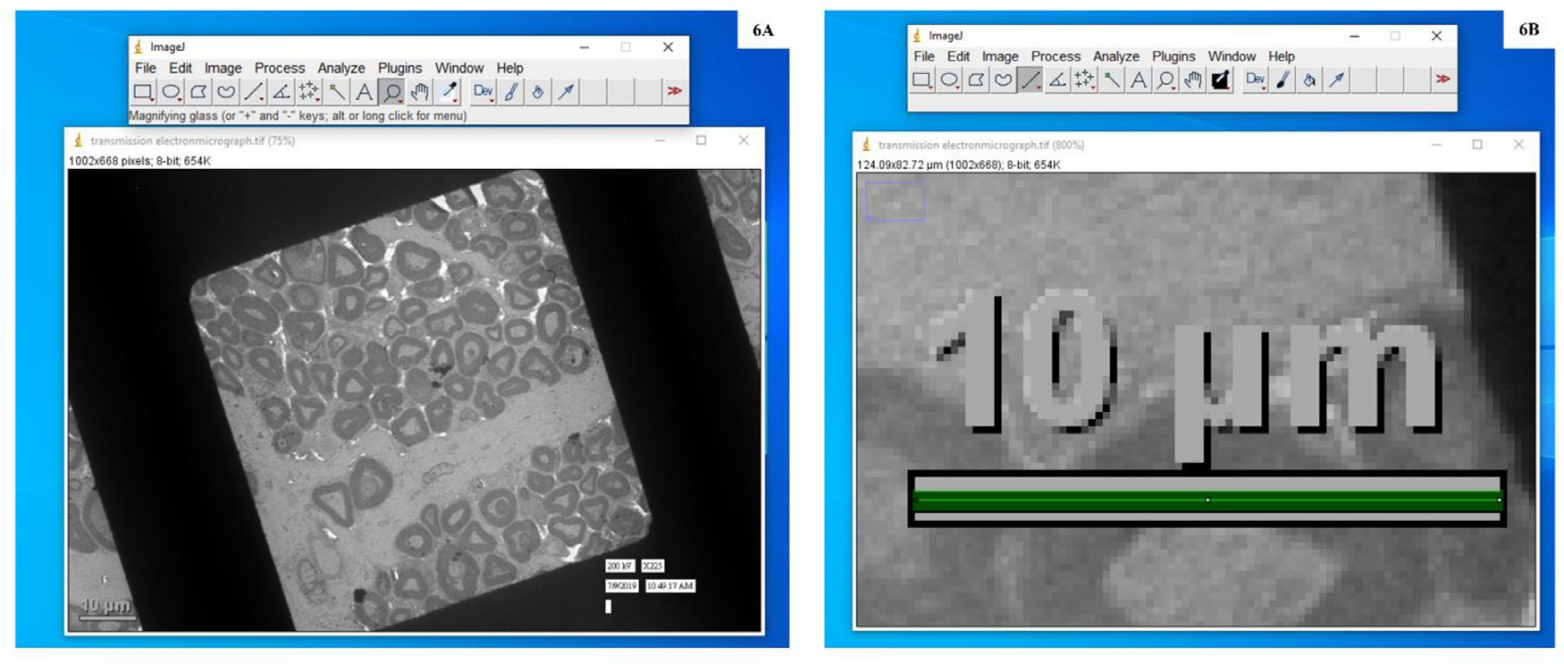
**A:** Screenshot to show the selection of the magnifying glass tool from the tool bar in the ImageJ window. **B:** Screenshot explains the magnification of the scale bar on the image, selection of straight line tool and drawing of straight line **(in green).**
7. Selected the ‘straight line’ tool from the tool bar in the ImageJ window and drew a straight line that would measure the length of the scale bar already present in electron micrographs (here it is 10 µm) (Fig. 6B).
8. Opened ‘Analyze’ menu and selected ‘Set Scale’ (Fig. 7). A dialogue box is opened in which the distance of pixels is based on the length of the line drawn over the scale bar present in the micrograph. Filled in the known distance (written on the scale bar e.g. 10) and unit of length (written on the scale bar e.g. µm). Then clicked OK (Fig. 8)

**Fig. 7:**
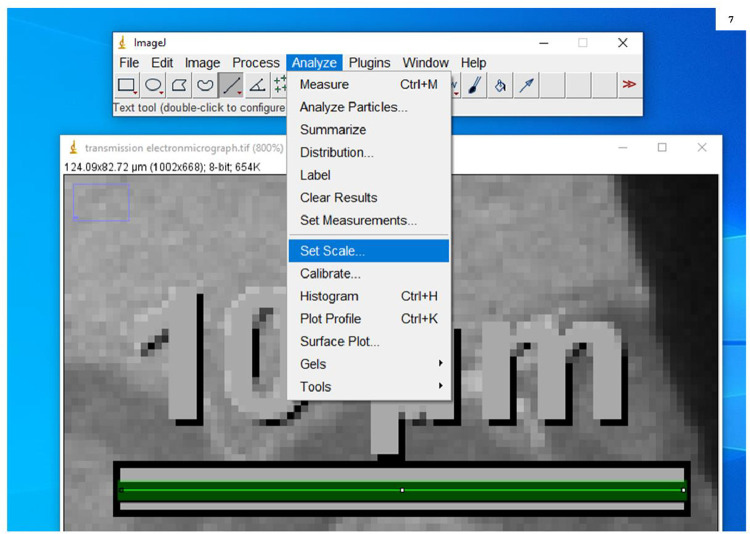
Screenshot to show the calibration of scale bar present in transmission electron micrograph with ImageJ software.

**Fig. 8:**
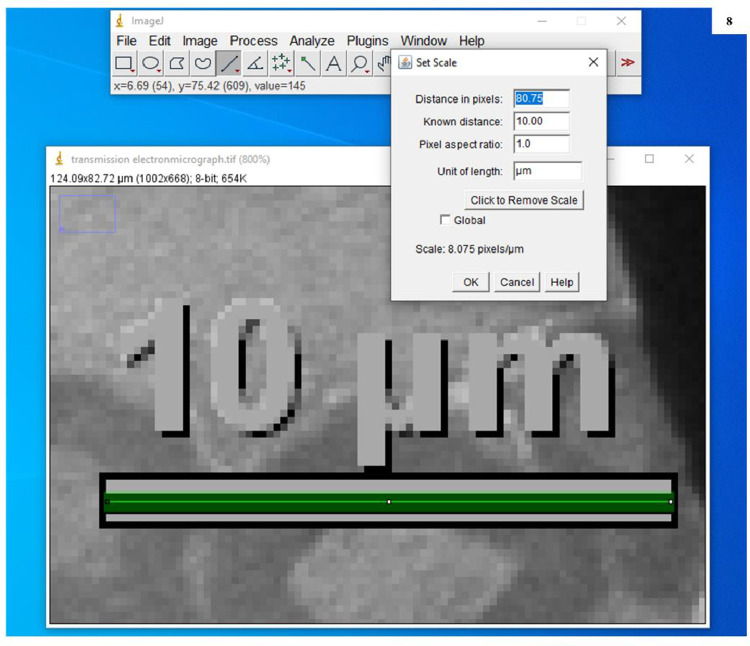
Screenshot to show the calibration of the embedded scale bar in the transmission electronmicrograph with ImageJ software.
9. Opened ‘Analyze’ menu and then clicked on ‘Measure’ or pressed on ctrl+*M* (Fig. 9A) to see the results (Fig. 9B), where length measured is the actual length of the scale bar.

**Fig. 9:**
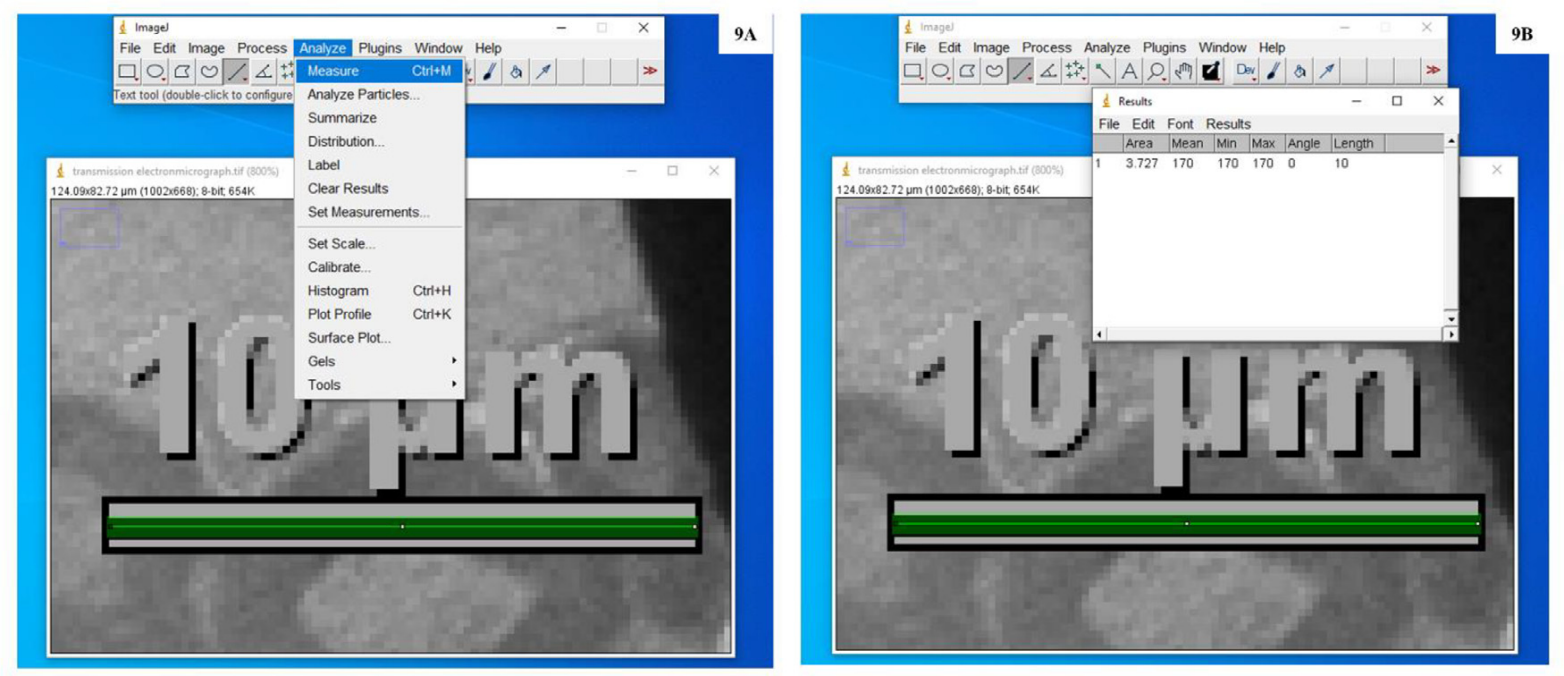
**A:** Screenshot to explain the measurement of length of calibrated scale bar present in transmission electron micrograph. **B:** Screenshot showing measurements of the scale bar present in transmission electron micrograph.
10. Drew four isotropic rays from the apparent centre of the transverse section of an individual nerve fibre forming two perpendicular test lines and measured distance from the centre of the nerve fibre to the inner and outer boundaries of the myelin sheath. These boundaries provided us the axon radius and nerve fibre radius, respectively (Fig. 10, Fig. 11, Fig. 12, Fig. 13).

**Fig. 10:**
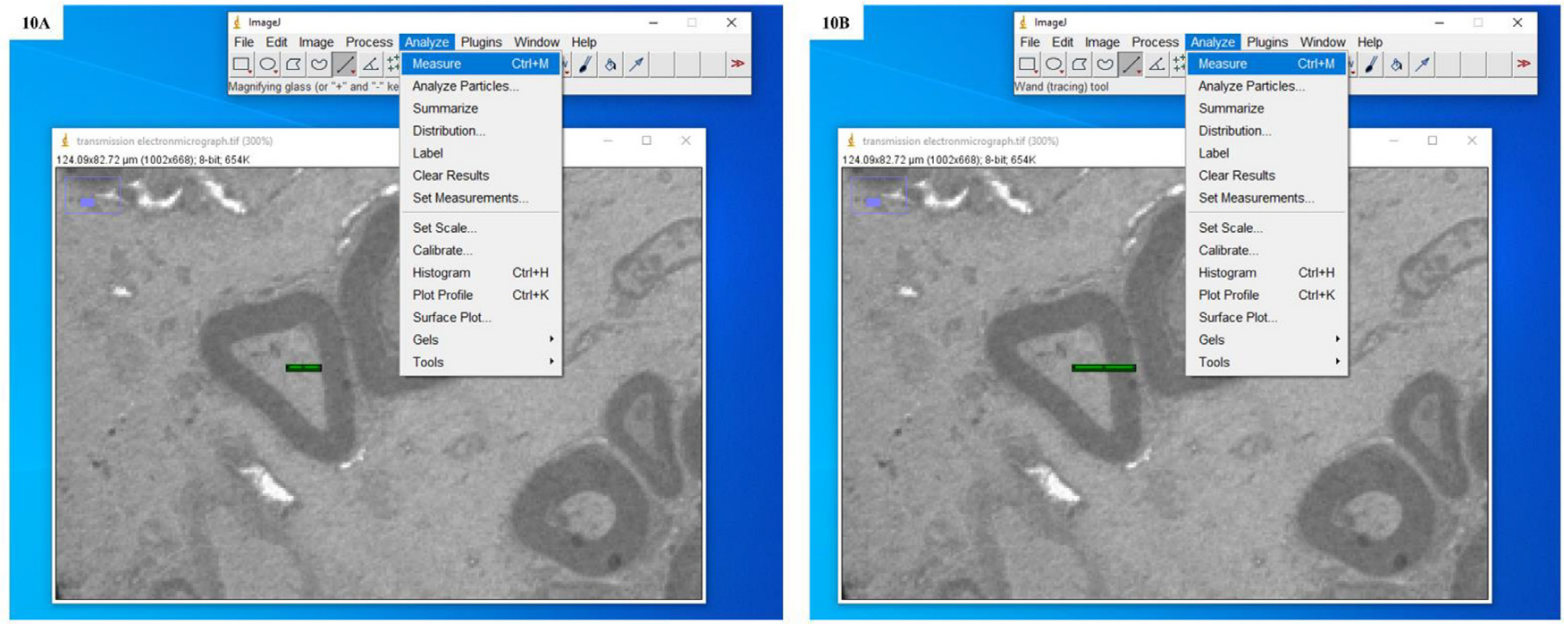
**A:** Screenshot showing the drawing of an isotropic ray and the measurement of distance from the apparent centre of the transverse section of an individual nerve fibre to the inner boundary of the myelin sheath. **B:** Screenshot showing the drawing of an isotropic ray and the measurement of distance from the apparent centre of the transverse section of an individual nerve fibre to the outer boundary of the myelin sheath.

**Fig. 11:**
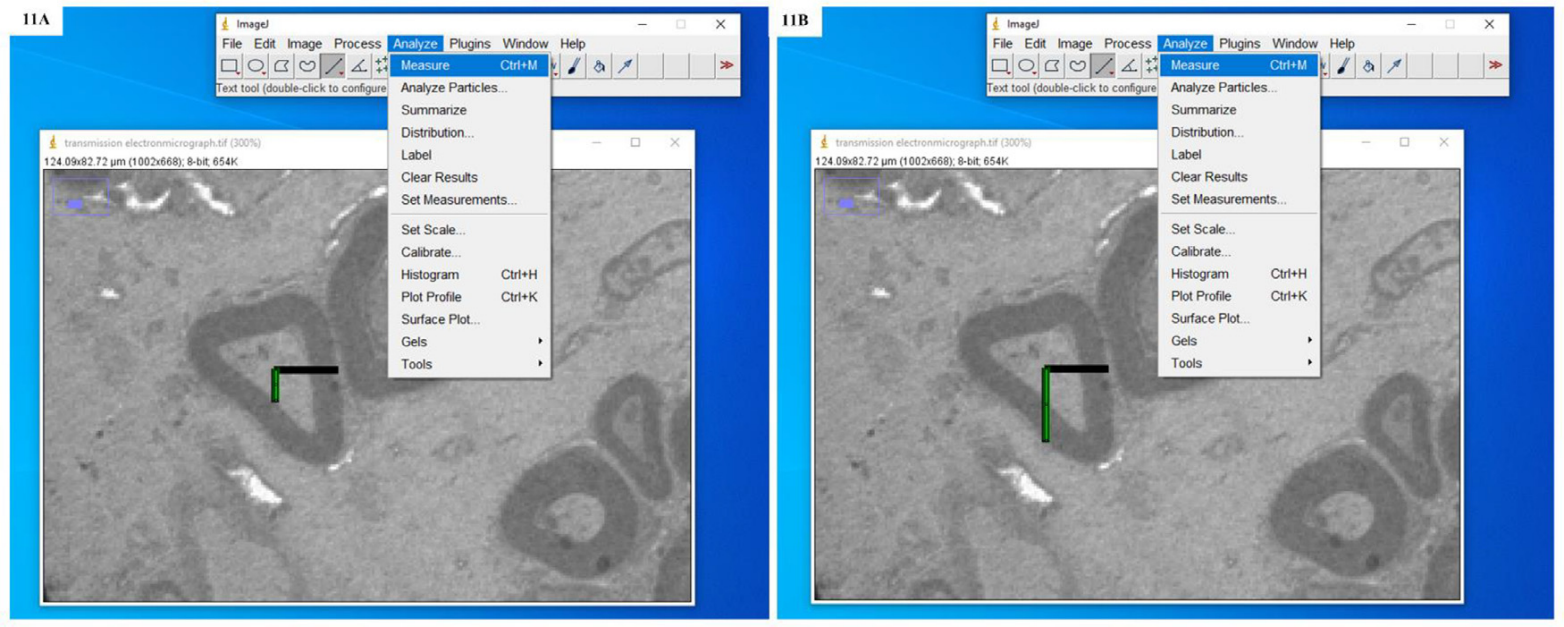
**A:** Screenshot showing the drawing of an isotropic ray and measurement of distance from the apparent centre of the transverse section of an individual nerve fibre to the inner boundary of the myelin sheath. **B:** Screenshot showing the drawing of an isotropic ray and measurement of distance from the apparent centre of the transverse section of an individual nerve fibre to the outer boundary of the myelin sheath.

**Fig. 12:**
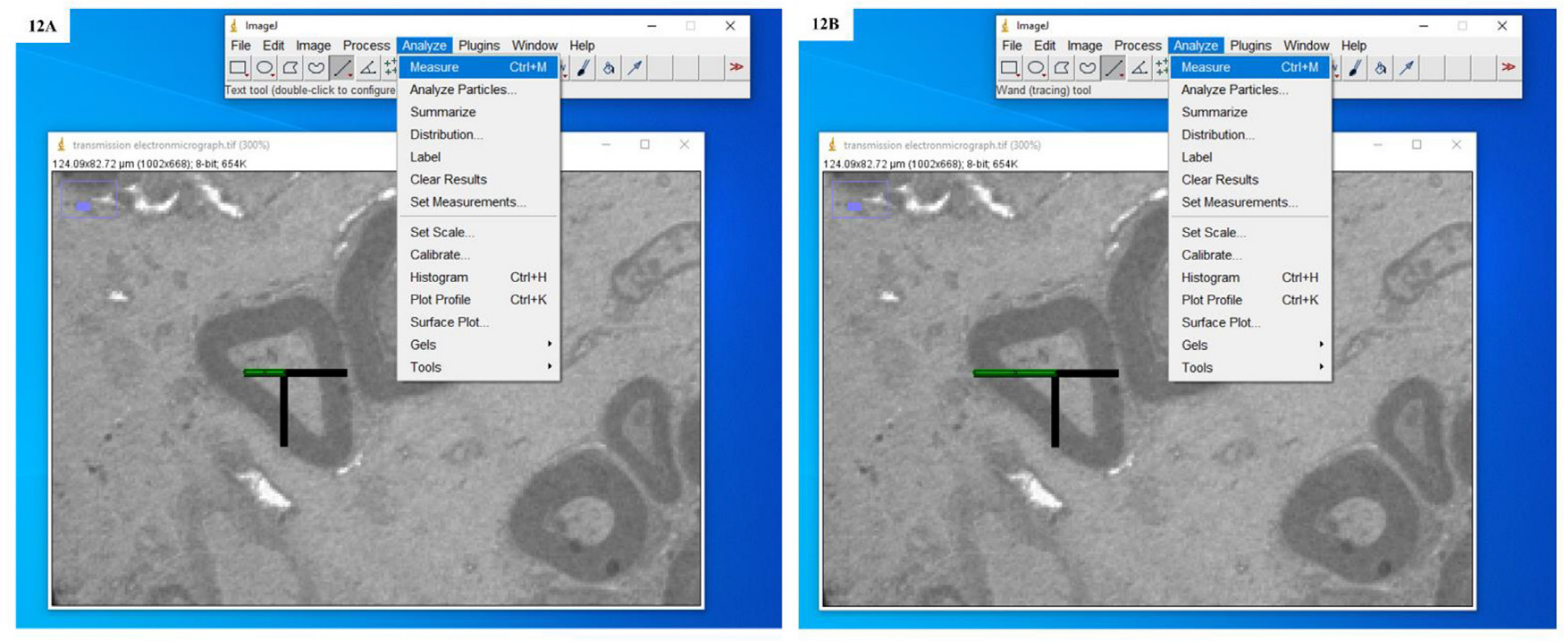
**A:** Screenshot showing the drawing of an isotropic ray and measurement of distance from the apparent centre of the transverse section of an individual nerve fibre to the inner boundary of the myelin sheath. **B:** Screenshot showing the drawing of an isotropic ray and measurement of distance from the apparent centre of the transverse section of an individual nerve fibre to the outer boundary of the myelin sheath.

**Fig. 13:**
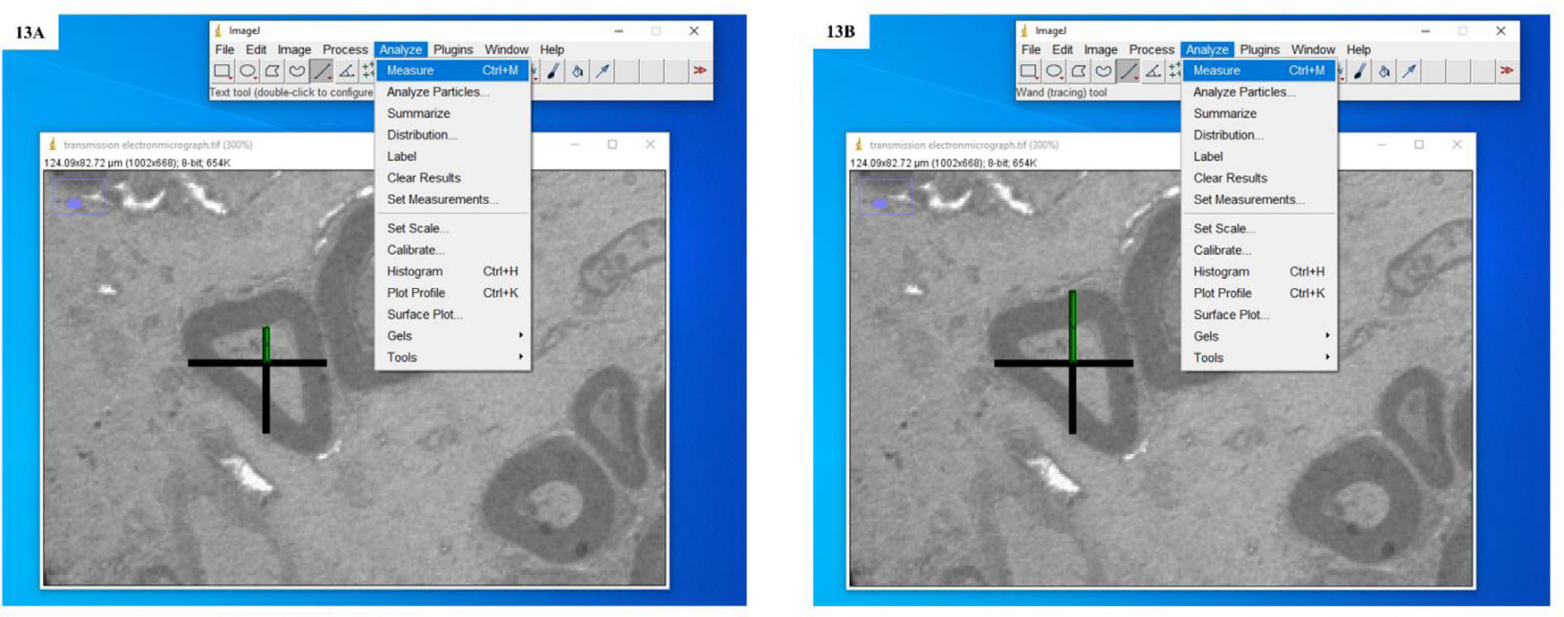
**A:** Screenshot showing the drawing of an isotropic ray and measurement of distance from the apparent centre of the transverse section of an individual nerve fibre to the inner boundary of the myelin sheath. **B:** Screenshot showing the drawing of an isotropic ray and measurement of distance from the apparent centre of the transverse section of an individual nerve fibre to the outer boundary of the myelin sheath.
11. Opened File and saved the results (Fig. 14).

**Fig. 14:**
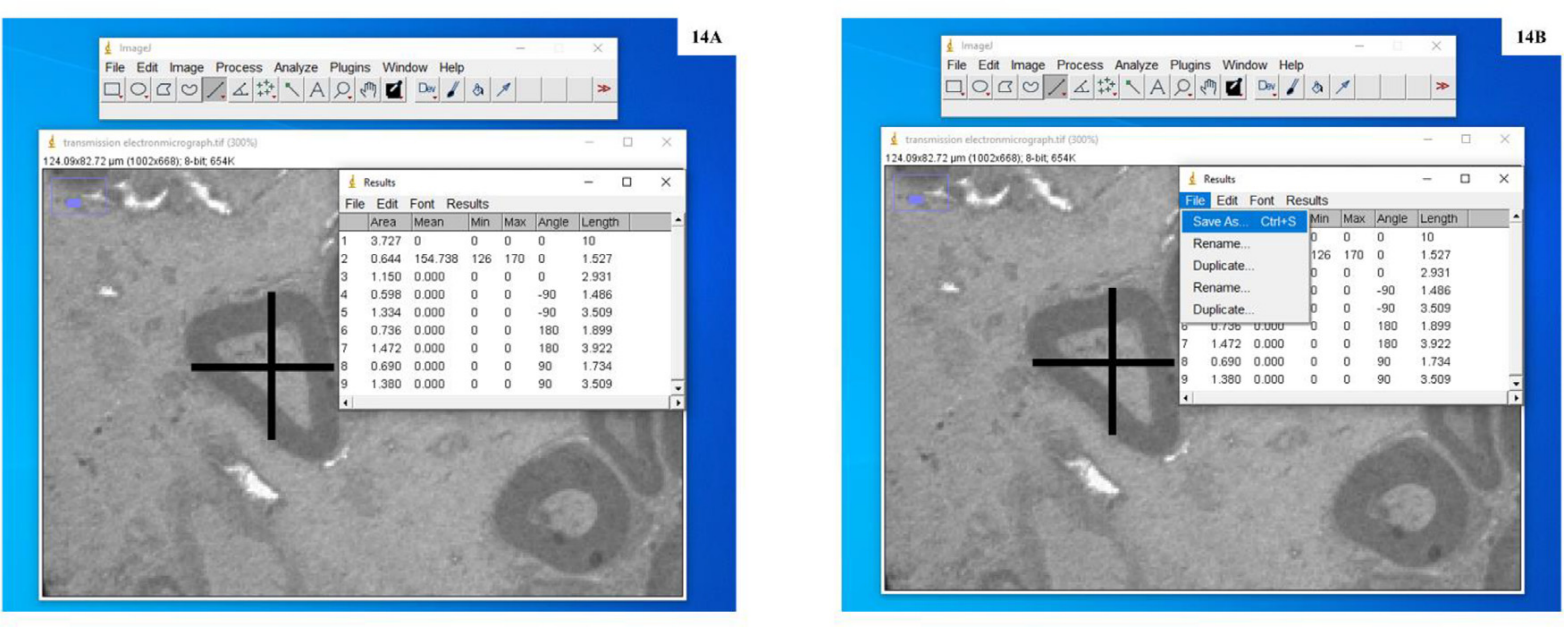
**A:** Screenshot showing the results generated after drawing four isotropic rays as two perpendicular test lines from the apparent centre of the transverse section of an individual nerve fibre measuring the distance from the centre of the nerve fibre to the inner and outer boundaries of the myelin sheath. **B:** Screenshot shows the saving of the results.
12. Calculated the area of the inner (occupied by axoplasm) and outer (axoplasm and myelin sheath) boundaries using the formula: *a*=πr^2^, where ‘r’ is the mean of the measured radii of the line drawn from the centre to the inner or outer boundary of the transverse section [2].
13. Calculated the diameters as 2r and myelin thickness as outer radius minus the inner one [7].
14. Calculated the G-ratio by dividing the axon diameter by the diameter of the whole nerve fibre.

### Note

**Fig. 15:**
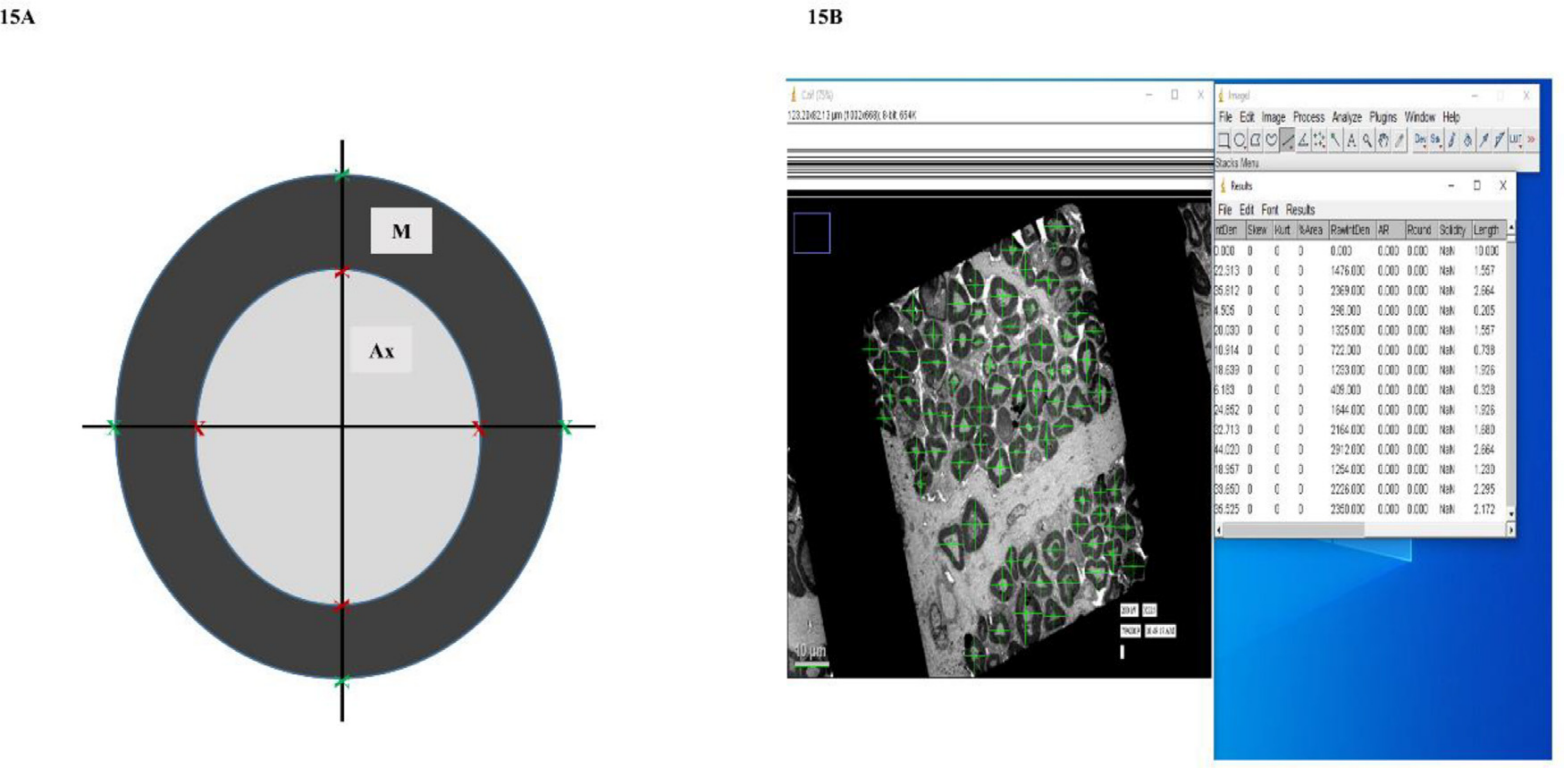
**A:** Schematic diagram representing a myelinated axon. The straight lines drawn in the figure were used to estimate the various morphometric parameters in the ImageJ software. Ax and M represent axon and myelin, respectively. Red and green Xs indicate the extent of inner (axon) and outer (myelin sheath) boundary of myelinated nerve fibre respectively. **B:** Screenshot of the captured digitised image of ultrathin section in one square of the copper mesh grid (left) and the list of parameters in use in the ImageJ software during image analysis (right).

Please go through Fig. 15A and B representing a myelinated axon and screen capture of the digitised image of ultrathin section in one square of the copper mesh grid (left), the list of parameters in use on ImageJ software during image analysis morphometry (right), respectively from our published research article based on the above method [5].

## CRediT authorship contribution statement

**Punit Kumar:** Conceptualization, Methodology, Investigation, Visualization, Writing – original draft. **Saroj Sharma:** Methodology. **Charanjeet Kaur:** Methodology, Investigation. **Indra Pal:** Investigation. **Daya Nand Bhardwaj:** Resources. **Tapas Chandra Nag:** Writing – review & editing. **Tara Sankar Roy:** Conceptualization, Supervision, Funding acquisition, Project administration, Writing – review & editing. **Tony George Jacob:** Conceptualization, Supervision, Project administration, Writing – review & editing.

## Declaration of Competing Interest

The authors declare that they have no known competing financial interests or personal relationships that could have appeared to influence the work reported in this paper.

## Data Availability

Data will be made available on request.
